# Measuring satisfaction with orthotic and prosthetic devices

**DOI:** 10.1007/s11136-026-04366-x

**Published:** 2026-08-07

**Authors:** Callie E. Tyner, Aaron J. Boulton, Jerry Slotkin, Pamela A. Kisala, Ross Zafonte, W. Lee Childers, Christopher L. Dearth, Josef Butkus, David S. Tulsky

**Affiliations:** 1 Center for Health Assessment Research and Translation, University of Delaware, Newark, DE USA; 2 Departments of Physical Medicine and Rehabilitation and Anesthesiology and Perioperative Medicine, University of Missouri School of Medicine, Columbia, MO USA; 3 Department of Rehabilitation Medicine, Center for the Intrepid, Brooke Army Medical Center, JBSA Ft. Sam Houston, San Antonio, TX USA; 4 Extremity Trauma and Amputation Center of Excellence, Defense Health Agency, Falls Church, Antonio, VA USA; 5 Department of Surgery, Uniformed Services University of the Health Sciences, Bethesda, MD USA; 6 Walter Reed National Military Medical Center, Bethesda, MD USA; 7 Departments of Physical Therapy and Psychological and Brain Sciences, University of Delaware, Newark, DE USA

**Keywords:** Patient reported outcome measures, Quality of life, Psychometrics, Prostheses and implants, Orthotic devices, Amputees

## Abstract

**Purpose:**

To develop a measure of satisfaction with orthoses and prostheses for the Limb Injury Measurement Battery for Quality of Life (LIMB-QOL).

**Methods:**

Items were developed to assess an individual’s satisfaction with, and emotional and physical assimilation of, an orthosis and/or prosthesis. Items covered topics of satisfaction with device performance and aesthetics as well as user adaptation to a device, including usage, fit, and comfort. A pool of 65 items was administered to 471 individuals in the LIMB-QOL field testing/calibration study. Item response theory was used to evaluate and calibrate the new items. Test–retest reliability after 1–2 weeks was evaluated in a subsample.

**Results:**

From the original pool, 39 items were removed for content and 11 items were removed due to differential item functioning by type of injury/limb loss and device (orthosis vs. prosthesis). Following psychometric analysis, 15 items were calibrated as an item bank. These items exhibited acceptable internal consistency (α = 0.95) and fit to a unidimensional model. The final 15-item bank has strong psychometric properties (test–retest reliability α = 0.79), can be administered as a computerized adaptive test or 7-item short form, and is applicable to upper and lower injuries, limb loss and reconstruction, and orthotic and prosthetic device users.

**Conclusion:**

The LIMB-QOL Satisfaction with Orthosis/Prosthesis is a reliable measure, with evidence for validity in individuals with a variety of limb-affecting injuries and sudden-onset illnesses, and thus can be used broadly following major extremity trauma, and would be ideal for large-scale monitoring of QOL outcomes, although further evaluation of the validity for individuals with dysvascular or degenerative conditions is needed.

**Electronic supplementary material:**

The online version of this article (https://doi.org/10.1007/s11136-026-04366-x) contains supplementary material, which is available to authorized users.

Quality of life (QOL) can be negatively affected by limb injury and loss, and dissatisfaction with orthotic or prosthetic devices prescribed to support rehabilitation and restoration of function is an important factor relevant for understanding health-related QOL (HRQOL) in these patients [1, 2]. Likewise, when individuals feel satisfied with their devices, this has been shown to be positively associated with HRQOL [2]. The impacts of prosthetic and orthotic device use on HRQOL for individuals with limb injuries and limb loss are complex, and include both physical components (e.g., functioning, activities of daily living [ADLs]) as well as psychosocial factors (e.g., body image, self-esteem) [3]. Comprehensive, standardized measurement of patients’ experiences with prosthetic and orthotic devices are important to evaluate treatment outcomes and perform comparative research [4, 5]. Developing a patient reported outcome measure (PROM) of prosthetic and orthotic device satisfaction for the Limb Injury Measurement Battery for Quality of Life (LIMB-QOL) [6] is the goal of this project.

For many individuals who experience major extremity injuries, prosthetic and/or orthotic devices are crucial for rehabilitation, whether limb loss and/or limb preservation/reconstruction are involved [7–11]. Prosthetic devices can be myoelectric, body powered, or cosmetic for the upper limb and passive or powered system [12, 13] or cosmetic devices for the lower limb. Each type of prosthesis serves different functions and provides different benefits [14–19]. Orthotic devices are used when the injured part of the limb is still present to provide support, as they can align, prevent, or correct limb pathologies, or aid return to function [11, 20, 21]. Orthoses can be used on the intact leg of individuals following unilateral lower limb loss [22], and have been used successfully in people with complex limb injuries to return to high levels of activity, including military service [11, 23, 24]. Some individuals have multiple devices for specific tasks/settings [25, 26]. Prosthetic devices that involve a socket as the interface between the user of the device have many different design considerations (e.g., prosthetic shape, suspension, and terminal device [14–16]), although osseointegration is becoming more widely available as a method to directly connect the prosthesis to the amputated bone [27–30]. Orthotic devices have many design considerations as well, including the intended purpose (e.g., support, protection, immobilization), the device’s function (i.e., dynamic, static, neurophysiologic), and physical factors (e.g., weight, material, appearance, ease of use) [31].

Having and using a prosthetic device has been found to be associated with higher HRQOL [32–35]. These devices may reduce experience of secondary physical conditions, which are common for both limb loss and reconstruction [36], and can also reduce neuropathic symptoms when well-fitted [37], but their use may also be associated with development of complications (e.g., osteoarthritis, osteopenia and osteoporosis, and back pain are common for individuals with lower-limb loss who use prosthetic devices [38, 39]). The therapeutic benefits of device use for individuals with lower-limb injuries center on ambulation [7, 14, 15, 33, 34, 40, 41], with prosthetic device use also being positively associated with social activities and independent living in individuals with lower-limb loss [41, 42]. Similarly, in studies of lower-limb orthosis users, participants reported improved ambulation, fewer falls, and improved HRQOL [9, 43, 44], although pain and psychosocial challenges can persist despite successful orthotics training [45–47]. For individuals with upper-limb loss, self-care tasks and activities of daily living (ADLs) are a major focus of device prescription [16, 48–50]. Furthermore, research has shown that employment rates are higher for individuals with upper- and lower-limb loss who use a prosthesis [32, 51].

Despite these benefits, device rejection and abandonment are commonplace, and may limit rehabilitation gains of patients. For example, research shows that approximately half of patients with lower-limb loss are dissatisfied with their prosthetic devices [1, 7, 38, 52], though device satisfaction is higher in certain contexts. One study reported that 75% or more of a sample of U.S. Service members with combat-related lower-limb loss reported being satisfied with the fit, weight, durability, and ease of putting on their prostheses, although many in this study reported dissatisfaction with aspects of comfort (e.g., negative impact on clothing, skin abrasion/irritation, and pain) [2]. In a pilot survey of 43 U.S. Veterans with traumatic amputation, all reported one or more problems with their prosthetic device [53]. A recent review of the literature [7] identified that pain [33], risk of falling [29, 30, 54], and mood factors [48] were negatively associated with prosthesis use for individuals with lower-limb loss, which echoes challenges identified in earlier reviews [38]. Despite these common concerns, many individuals with lower-limb loss in another study were still satisfied enough to use their prosthesis much of the day [54].

The limitations of prosthetic devices for upper extremity limb loss have also been widely acknowledged [55], with contextual and individual factors affecting device adoption and use [56]. Upper-limb prosthesis rejection rates of 18–26% have been observed, with limited functionality and discrepancy between need and device capabilities given as common reasons [32, 57]. Delays in prosthesis availability are associated with lower acceptance [58]. Patient expectations appear to play a role in perceived outcomes, and psychoeducation has been proposed as a way to reduce device abandonment [59, 60]; thus, measures of satisfaction are needed to provide an assessment of the impact of novel device designs and technologies for customizations (e.g., 3D printing, robotics, haptics).

## Approach to item development

Given the need for standardized measures that can be used broadly to understand the HRQOL outcomes following majority extremity limb loss and reconstruction [4, 5], our team aspired to develop a measure for the LIMB-QOL on device use that would be applicable to: (1) both prosthesis and orthosis users, (2) individuals with limb loss and those whose limbs were treated with surgical reconstruction (such as preservation, reconstruction, or salvage procedures), (3) both upper and/or lower limb involvement, and (4) different levels/extents of limb loss or disability, including injuries or limb loss involving multiple limbs. There is currently only one measure that meets these requirements (Table 1), which is the Satisfaction with Device module of the Orthotics & Prosthetics User Survey [OPUS; 62], although the OPUS items use a present tense format that differs from the rest of LIMB-QOL. Based on our prior qualitative work [77] and input from experts, we sought to develop additional items covering multiple aspects of how prostheses and orthoses may affect HRQOL. These included their impacts on participation, physical functioning, ADLs, mood, behaviors related to device usage and interaction with clothing, perception of durability and utility, and emotional reactions such as gratitude, acceptance, satisfaction, and comfort in public. Importantly, we avoided duplicating item content covered by other LIMB-QOL measures, such as pain, mobility, upper-extremity functioning, body image, self-esteem, and vocational impacts [6]. We also endeavored to develop items that would be applicable to users of future advanced technology (e.g., robotics, haptics [78–81]), given the rapid advancement in this field and our desire for the LIMB-QOL measures to maintain long-term relevance. Last, consideration was given to develop items that would be applicable across the full range of abilities/functioning; for example, being equally applicable to a young Military Service member with single lower-limb loss seeking to return to active duty as it would be to an older individual with multiple surgically treated limbs from a motor vehicle accident with a rehabilitation goal of independence in ADLs.

**Table 1:** Existing self-report measures considered related to satisfaction with prosthetic and/or orthotic devices

Measure name	References	Target population	Description
Orthotics and prosthetics user survey (OPUS)	[61]	Both prosthesis and orthosis users	The OPUS contains five modules, including two modules that are relevant to this project: HRQOL (23 items) and Satisfaction with Device and Services (21 items).^a^ Scoring is based on Rasch analysis keyform lookup table
Prosthetic evaluation questionnaire (PEQ)	[62, 63]	Unilateral lower-limb loss, prosthesis users	The PEQ contains 82 questions, 42 of which are grouped into nine validated scales across the areas of prosthesis function, mobility, psychosocial experience, and well-being. Responses are collected via a visual analogue scale and scoring is based on measuring the response in millimeters from the left endpoint of the line (1–100). Scoring is the average of the responses in each scale
Prosthetic limb users survey of mobility (PLUS-M)	[64–68]	Unilateral or bilateral lower-limb loss prosthesis users	The PLUS-M is a measure of mobility that is available in short form (7- or 12-item) and computer adaptive test (CAT) formats, and scoring renders T-scores
Quebec user evaluation of satisfaction with assistive technology (QUEST)	[69–72]	Any user of assistive technology	The QUEST 2.0 has 12 items on the topics of satisfaction with an assistive device (8 items) and associated services (4 items). Response options range from 1–5, with 1 = “not satisfied at all” to 5 = “very satisfied.” The measure yields a total score as well as separate scores for satisfaction with device and services (calculated by averaging responses to the items)
Satisfaction with the prosthesis questionnaire (SATPRO)	[73]	Lower-limb loss, prosthesis users	The SATPRO is a 15-item measure of satisfaction with use of the prosthesis. It uses a 4-point scale from 1 = “totally agree” to 4 = “totally disagree”; items are summed to generate the total score
Trinity amputation and prosthesis experience scales, revised (TAPES-R)	[74, 75]	Limb loss, prosthesis users only	The TAPES includes three psychosocial adjustment subscales (general adjustment, social adjustment, and adjustment to limitations), which are five questions each. Responses range from 1 = “strongly disagree” to “4 = “strongly agree,” and scoring is the mean divided by the number of answered items in each subscale

^a^ = The OPUS Satisfaction with Device scale contains content that is the most similar to the LIMB-QOL item bank developed in this project, although the items differ in tense and response set. Four items covering content similar to OPUS items were included in field testing (on the topics of fit, ease of putting on, appearance, and durability of the device), although only one of these was included in the final, calibrated bank (OrthProsth002). See Heinemann et al. [76] for a recent review of PROMs for ankle–foot orthosis users

## Methods

### Participants

The LIMB-QOL field testing sample consisted of 603 individuals with a history of majority extremity injury or limb loss [6]; of these, 471 individuals who reported having an orthotic and/or prosthetic device(s) were administered items from the Satisfaction with Orthosis/Prosthesis item pool. Other inclusion criteria for enrollment were as follows: (1) Injury to one or more extremities that necessitated surgical intervention—any qualifying limb that was preserved following surgery was further required to (1a) impact at least two bodily systems, (1b) result in a functional deficit or be reported as requiring preservation or salvage surgery, and (1c) be injured following a traumatic or sudden-onset event; (2) At least 18 years of age; (3) Less than 65 years of age at time of enrollment; (4) Able to read, speak, and understand English.

### Study procedures

Nine medical facilities—three military and six civilian medical treatment centers—were responsible for participant recruitment into the study. Protocols were approved by IRBs at each site. Once enrolled, participants provided responses to items during approximately 2 h telephone interviews, administered by trained data collectors. Follow-up interviews occurred one year later for a subset of the sample, to collect data to estimate 1- to 2-week test–retest reliability and convergent validity coefficients.

### Item pool and validation measures

The Satisfaction with Orthosis/Prosthesis item pool was originally comprised of 65 items that were newly written to assess an individual’s satisfaction with and emotional and physical assimilation of an orthosis and/or prosthesis. Tulsky et al. [6] describes the multi-stage process used to develop the new LIMB-QOL item pools, involving item writing and expert review, as well as cognitive interviews with stakeholders. All items in the pool utilized a 5-category response option format (Never; Rarely; Sometimes; Often; Always) and asked participants about device satisfaction occurring “In the past 7 days.” Higher item scores were associated with greater satisfaction. For participants with multiple devices, they were instructed to respond to items thinking about the device or devices that they typically use.

To assess convergent validity of the final item bank, study participants also completed additional measures at the 1-year follow-up, including the three Psychosocial Adjustment subscales (General Adjustment, Social Adjustment, Adjustment to Limitation) of the TAPES-R instrument [74, 75] (Table 1), the Amputee Body Image Scale—Revised (ABIS-R) [82] and the 10-item Patient-Reported Outcomes Measurement Information System (PROMIS) Global Health [83] measure of physical and mental health. The TAPES-R has shown moderate correlation with prosthesis satisfaction-related factors, such as daily wearing time for Veterans with unilateral upper-limb loss [84], and has favorable psychometric properties [85, 86]. Likewise, the ABIS-R—a measure of body image specific for prosthetic users with amputation—and the PROMIS Global Health measures have shown favorable psychometric properties in this population [87–89]. We hypothesized that the new LIMB-QOL items would be moderately correlated (> 0.40) with these related, but conceptually distinct, measures.

### Data analysis

The primary objective of analyses was to narrow the pool of potential items to create a unidimensional, reliable, and valid measure of individual’s satisfaction with their orthotic and/or prosthetic device(s). The graded response model (GRM [90]), an item response theory (IRT) model for polytomous item response data, was used as the focal measurement model. Several psychometric procedures arising from the classical test theory and IRT frameworks were conducted to ensure GRM assumptions were met. After all psychometric analyses were conducted, a small number of items were removed—typically 1–5 items at a time—and analyses re-run iteratively to generate new item- and test-level statistics and make further revisions to the item pool or proceed to differential item function (DIF) analyses. Once the item set was finalized (i.e., all psychometric and DIF criteria were satisfied), we further evaluated test–retest reliability, construct validity, and abbreviated measure administration. As a result of the extensive nature of the analytic approach, we provide only a high-level summary of the analytic plan here; readers are referred to Boulton et al. [91] for in-depth discussion of each analytic procedure and accompanying decision-making criteria used to determine item exclusion at the end of each iteration.

There are three primary assumptions underlying the GRM: monotonicity of the item response functions, unidimensionality, and local independence. Mokken scale analyses [92] were used to visually assess monotonicity of the response category functions; item- and test-level H scalability coefficients [93] were also computed from the Mokken analyses (H > 0.5 was indicative of “strong” scalability [94]). Multiple procedures were used to assess unidimensionality. Multidimensional scaling (MDS) [95] and item cluster analysis [96] diagrams were generated to evaluate unidimensionality graphically. Fit of a unidimensional confirmatory factor analysis (CFA) model—indicated by the following indices/thresholds: Root Mean Square Error of Approximation (RMSEA) < 0.08, Comparative Fit Index (CFI) and Tucker-Lewis Index (TLI) > 0.90, Standardized Root Mean Square Residual (SRMR) < 0.06—provided further evidence of unidimensionality, as well as omega hierarchical reliability index values > 0.7 [97]. Local independence was confirmed by inspecting pairwise residual item correlation from the CFA, ensuring values were < 0.20 [98]. Once items that violated GRM assumptions were removed and criteria satisfied, GRM item parameters were estimated and DIF analyses were conducted. IRT-based ordinal logistic regression analyses were used to evaluate DIF for several subgroups in the calibration sample: age (< 40 vs. 40 +), military status (civilian vs. active-duty/reservist/veteran), injury location (lower limb only vs. upper limb/both upper and lower limbs), limb loss status (any major extremity amputated due to injury vs. no amputations), and time since injury (< 3 years vs. 3 + years). Items were flagged for DIF if the McFadden pseudo R^2^ exceeded .01, and were further inspected using graphical and numerical output, and generally removed; items flagged for DIF but deemed essential for content coverage or negligibly influential on item bank scores were in limited cases retained but excluded from inclusion on short forms.

Following these analyses, the item bank was considered final and short form and CAT formats were developed and compared to the full item bank for score distribution, reliability, and correlation. Short forms were constructed by selecting items with large discrimination values (i.e., those items providing large contributions to total scores) and broad content coverage as reflected in diverse item locations [91]. Three CAT specifications (two fixed-length and one variable-length CAT) were examined, as described below. Test–retest reliability was quantified using the intraclass correlation coefficient (ICC); ICC values ≥ 0.75 or .90 were considered good or excellent, respectively [99].

## Results

### Participants

Demographic and injury characteristics of the subsamples that completed Satisfaction with Orthosis/Prosthesis items are shown in Table 2. At baseline assessment, 471 individuals owned one or more prosthesis or orthosis, and thus provided the responses used for item pool refinement and scale setting (i.e., the calibration sample). At the 1-year follow-up assessment, 145 participants completed items from the calibrated item bank and validation measures. Finally, 145 participants completed the 1- to 2-week retest assessment (Table 2, footnote, regarding differences between the 1-year follow-up and retest samples). The subsample characteristics were largely stable over time, with majorities of participants who reported as being (a) male (77–82%), (b) non-Hispanic (84–86%), (c) white (78–80%), and (d) civilian (41–53%) or Veteran (35–46%), with injuries that were (e) lower limb(s) only (70–73%), (f) resulting from a traumatic event (80–85%), and (g) with limb loss (78–97%). The majority of the samples used one or more prosthetic device (65–88%), some used orthotic devices only (0–21% range), and a limited number used both prosthetic and orthotic devices (9–13%). Frequency of device use (days per week) is shown in Table 3.

**Table 2:** Demographic and injury characteristics of participant subsamples

	Calibration sample (*n* = 471)		Validation study sample (*n* = 145)^d^		Test–retest study sample (*n* = 145)	
	M	SD	M	SD	M	SD
Age	41.0	12.9	43.6	12.9	42.2	12.7
Time since injury (Years)	9.5	9.2	12.3	10.7	11.8	10.8

	n	%	n	%	n	%
Gender						
Male	380	80.7	112	77.2	119	82.1
Female	89	18.9	33	22.8	26	17.9
Other	1	0.2	0	0.0	0	0.0
Do not wish to provide	1	0.2	0	0.0	0	0.0
Ethnicity						
Non-hispanic	403	85.6	124	85.5	122	84.1
Hispanic	66	14.0	21	14.5	23	15.9
Do not wish to provide	2	0.4	0	0.0	0	0.0
Race						
American Indian/Alaskan Native	3	0.6	1	0.7	1	0.7
Asian	5	1.1	2	1.4	3	2.1
Black or African American	46	9.8	9	6.2	9	6.2
Native Hawaiian or Other Pacific Islander	5	1.1	3	2.1	3	2.1
White	368	78.1	116	80.0	114	78.6
Other	26	5.5	7	4.8	8	5.5
Do not wish to provide	3	0.6	1	0.7	1	0.7
Multiple	15	3.2	6	4.1	6	4.1
Injury location						
Upper	62	13.2	20	13.8	21	14.5
Lower	345	73.2	102	70.3	106	73.1
Both upper and lower	64	13.6	23	15.9	18	12.4
Limb status^a^						
Limb loss/Amputated	370	78.6	140	96.6	119	82.1
Surgically treated	101	21.4	5	3.4	26	17.9
Device use status						
Prosthetic device user	308	65.4	127	87.6	108	74.5
Orthotic device user	101	21.4	0	0.0	24	16.6
Both prosthetic and orthotic device user	62	13.2	18	12.4	13	9.0
Trauma status^b^						
Traumatic	399	84.7	117	80.7	123	84.8
Non-traumatic	72	15.3	28	19.3	22	15.2
Military status						
Active-duty^c^	106	22.5	18	12.4	27	18.6
Veteran	168	35.7	64	44.1	67	46.2
Civilian	197	41.8	63	43.4	51	53.2

Due to rounding, percentages within categories may not add to 100

^a^ = Limb status of “Limb loss/Amputated” is defined as the number of people in the sample who had experienced the loss of at least one major extremity due to a qualifying cause, and “Surgically Treated” indicates the presence of no limb loss/amputations

^b^ = Trauma status of “Traumatic” is defined as the number of people in the sample who had experienced at least one qualifying major extremity injury having occurred due to a traumatic cause, with “Non-Traumatic” indicating no limb injuries were due to a traumatic cause

^c^ = Includes military reservists

^d^ = Validation study sample size references those who completed both the LIMB-QOL Satisfaction with Orthosis/Prosthesis short form and TAPES-R, see Table S1 for demographic information on participants who completed other validation measures. While identical numerically, the subsample of 145 individuals who completed the validation study does not overlap completely with the 145 individuals who completed the test–retest assessment

**Table 3:** Average T-score by device use frequency at baseline

	How many days per week do you typically use your prosthetic device or devices?			How many days per week do you typically use your orthotic device or devices?		
	n	M	SD	n	M	SD
Daily (6 or 7 days/week)	321	52.67	8.68	116	48.14	8.07
3–5 days/week	29	44.91	7.21	16	45.88	9.61
Less than 3 days/week	6	32.61	9.67	17	44.79	9.31
Not at all	14	34.10	10.24	14	45.39	8. 66

Please refer to Table 2 for information on how many participants in the baseline sample were users of prostheses, orthoses, or both

## Item bank calibration and evaluation

Eleven iterations of item analyses were conducted and a majority of items were removed to create a final 15-item bank. The first seven iterations resulted in removal of 39 items prior to DIF testing. Predominant reasons for item removal during these iterations were content concerns (e.g., lack of conceptual fit with other items), low factor loading/item discrimination estimates, or local item dependence. At iteration 8, numerous items were flagged during DIF testing when comparing individuals with versus without limb loss; 8 items were removed due to DIF at this step. The next two rounds of item analyses led to the removal of 3 additional items. At the final iteration, all item analytic psychometric criteria were met and only 2 items were flagged for DIF. Although these items exceeded the effect size threshold (pseudo-R^2^ > 0.01) demarcating DIF, further evaluation indicated the impact of DIF was negligible, and the remaining 15 items were designated as the final item bank.

**Fig. 1:**
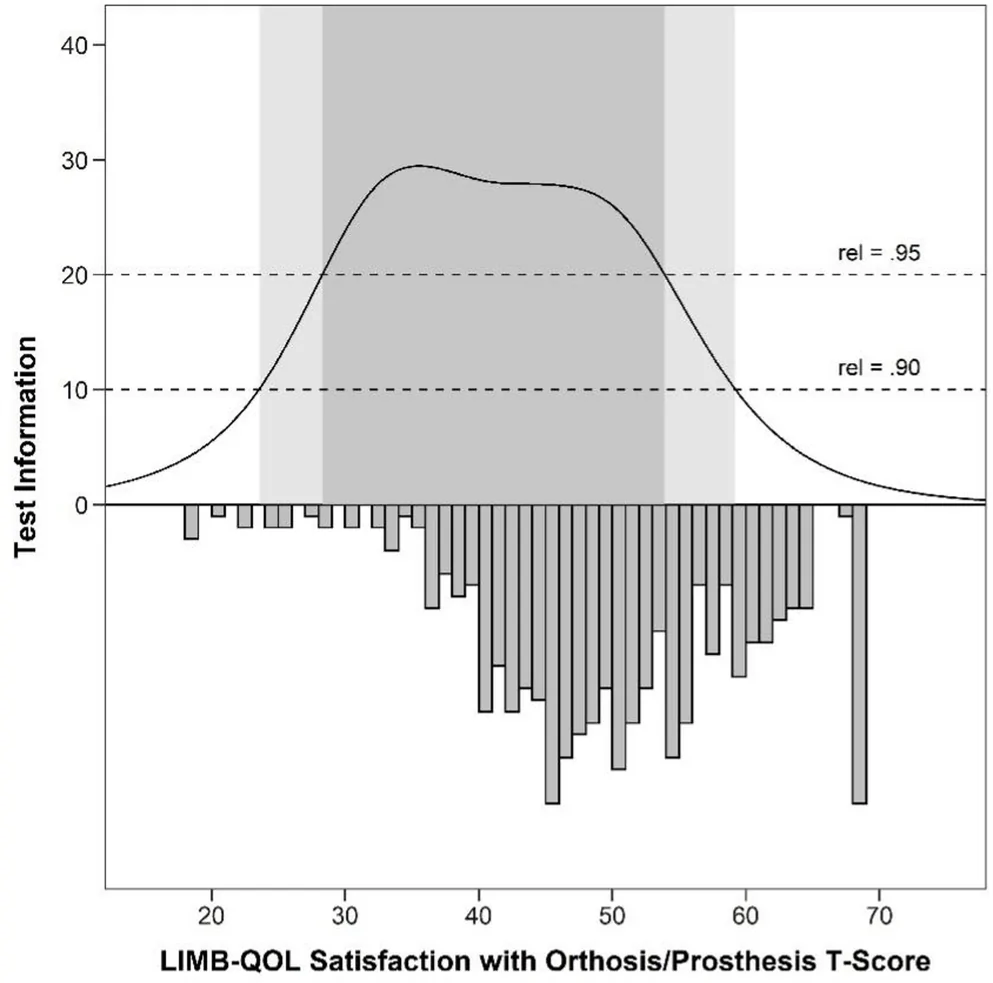
Test information and calibration sample score histogram for LIMB-QOL Satisfaction with Orthosis/Prosthesis. In the upper panel, test information is shown across the latent trait continuum. The dashed lines reflect classical reliability values of 0.90 and 0.95, and the boxes reflect the range of scores over which reliability exceeds 0.90 (light gray) or 0.95 (dark gray). The T-score distribution in the lower panel was largely normally distributed, with a slight ceiling effect (6%) at the maximum T-score of approximately 68

Internal consistency of the final 15 items was excellent (α = 0.95), and unidimensionality was supported by bifactor model-based reliability (ω_H_ = 0.85), single-factor CFA model fit [χ^2^ (90) = 286.208, CFI = 0.995, TLI = 0.995, RMSEA = 0.068, SRMR = 0.050), and exploratory factor analysis parameter estimates (highly correlated first 3 dimensions). Furthermore, all items merged into a single cluster with no obvious outliers apparent in the MDS visualization. Item response frequencies were negatively skewed (indicating high levels of device satisfaction within the sample), though all response categories contained at least 10 item responses. No gross violations of monotonicity were identified and the test- and item-level H coefficients exceeded thresholds (test H coefficient = 0.542; item H coefficients = 0.47 − 0.62). GRM discrimination and threshold parameter estimates are shown in Table 4. Item discrimination estimates ranged between 1.41 and 3.72 and threshold values ranged between -3.26 and 1.13. The item with the lowest discrimination value also exhibited significant item misfit [χ^2^ (57) = 106.708]; however, the item content was considered unique and central to the measure definition (orthprosth002: “I felt that my orthosis/prosthesis was durable”) and was therefore retained. All other item discrimination parameters were greater than 1.73. The distribution of T-scores (M = 50, SD = 10) for the final bank, computed using the expected a posteriori (EAP) method, are shown in Fig. 1. The score distribution is approximately normal with a very slight ceiling effect (5.6%, Table 4), with classical reliability exceeding .90 over 3 SDs of the score continuum.

**Table 4:** Item descriptive statistics and grade response model parameter estimates

Item ID	Item stem	Item descriptives				GRM item parameter estimates					Notes
		Mean	SD	% at min	% at max	Slope	T1	T2	T3	T4	
**orthprosth056**	**My orthosis/prosthesis improved my quality of life**	**4.2**	**1.1**	**4.3**	**40.5**	**3.3852**	**− 1.7737**	**− 1.4637**	**− 0.8016**	**− 0.0763**	**SF, RT**
orthprosth078	Wearing my orthosis/prosthesis improved my mood	3.6	1.3	8.1	24.0	2.1942	− 1.5856	− 1.1428	− 0.3465	0.5904	
orthprosth072	It felt normal to wear my orthosis/prosthesis	4.1	1.2	4.1	38.5	2.7070	− 1.9027	− 1.4318	− 0.7162	0.0115	
**orthprosth006**	**My orthosis/prosthesis helped me to participate in the activities I wanted to**	**4.0**	**1.1**	**4.1**	**35.5**	**3.7187**	**− 1.7321**	**− 1.3469**	**− 0.6307**	**0.0988**	**SF**
orthprosth007	My orthosis/prosthesis enabled me to do the physical activities I wanted to do	3.7	1.2	6.3	24.9	2.4927	− 1.7156	− 1.3036	− 0.4072	0.5029	
**orthprosth018**	**I was able to do everyday tasks while wearing my orthosis/prosthesis**	**4.0**	**1.1**	**4.0**	**35.5**	**2.5904**	**− 1.9389**	**− 1.5019**	**− 0.6446**	**0.1163**	**SF, RT**
orthprosth087	I felt comfortable wearing my orthosis/prosthesis in public	4.3	1.1	2.7	45.8	1.9266	− 2.4934	− 1.8189	− 1.0833	− 0.2816	
**orthprosth083**	**I was able to wear the clothing I wanted to while using my orthosis/prosthesis**	**3.9**	**1.3**	**6.1**	**34.3**	**1.6039**	**− 2.0890**	**− 1.4687**	**− 0.6917**	**0.2224**	**SF, RT**
**orthprosth058**	**I felt that my orthosis/prosthesis was like a part of my body**	**3.2**	**1.3**	**11.4**	**15.4**	**1.8499**	**− 1.4477**	**− 0.7550**	**0.0705**	**1.1248**	**SF**
orthprosth068	I felt that my life was better because of my orthosis/prosthesis	3.9	1.2	6.1	33.5	2.6572	− 1.6236	− 1.3903	− 0.6387	0.1661	
**orthprosth042**	**I was thankful for my orthosis/prosthesis**	**4.3**	**1.0**	**2.3**	**43.0**	**2.0325**	**− 2.5426**	**− 2.0128**	**− 1.0869**	**− 0.1608**	**SF**
**orthprosth073**	**I enjoyed using my orthosis/prosthesis**	**3.7**	**1.2**	**5.8**	**26.2**	**3.1338**	**− 1.6174**	**− 1.1232**	**− 0.3338**	**0.4400**	**SF, RT**
orthprosth002	I felt that my orthosis/prosthesis was durable	4.3	0.9	1.8	39.3	1.4131	− 3.2570	− 2.5940	− 1.5381	− 0.0098	
orthprosth024	My orthosis/prosthesis was helpful	4.4	0.9	2.3	46.9	3.5239	− 2.1623	− 1.7197	− 1.1432	− 0.2801	
orthprosth063	Once my orthosis/prosthesis was on, I preferred to keep it on	3.7	1.2	6.0	24.2	1.7346	− 2.0388	− 1.3119	− 0.4965	0.6519	

Context for all items was “In the past 7 days…”; Response set for all items was: 1 = Never / 2 = Rarely / 3 = Sometimes / 4 = Often / 5 = Always. T1-T4 = Item threshold estimates. SF = the items selected for the fixed-length short form (SF; known as SF7a; also indicated with boldface text); RT = items administered at the 1-year follow-up and 1–2 week retest assessments, known as SF4b. LIMB-QOL items and parameters copyright © 2026 David Tulsky and The University of Delaware. All rights reserved. Publication of item content, tables, parameters, or descriptive materials concerning the copyrighted instrumentsdoes not place the copyrighted instruments in the public domain and does not constitute permission to reproduce, adapt, distribute,k or otherwise use the copyrighted instrumentsexcept as expressly authorized. Measures should be accessed and used by sending a request to LIMB-QOL@udel.edu or through Assessment Center, the NIH Toolbox iPad application, the NIH PROMIS iPad application, or the REDCap instrument library [100–105]. Do not modify items without permission from the copyright holder

**Fig. 2:**
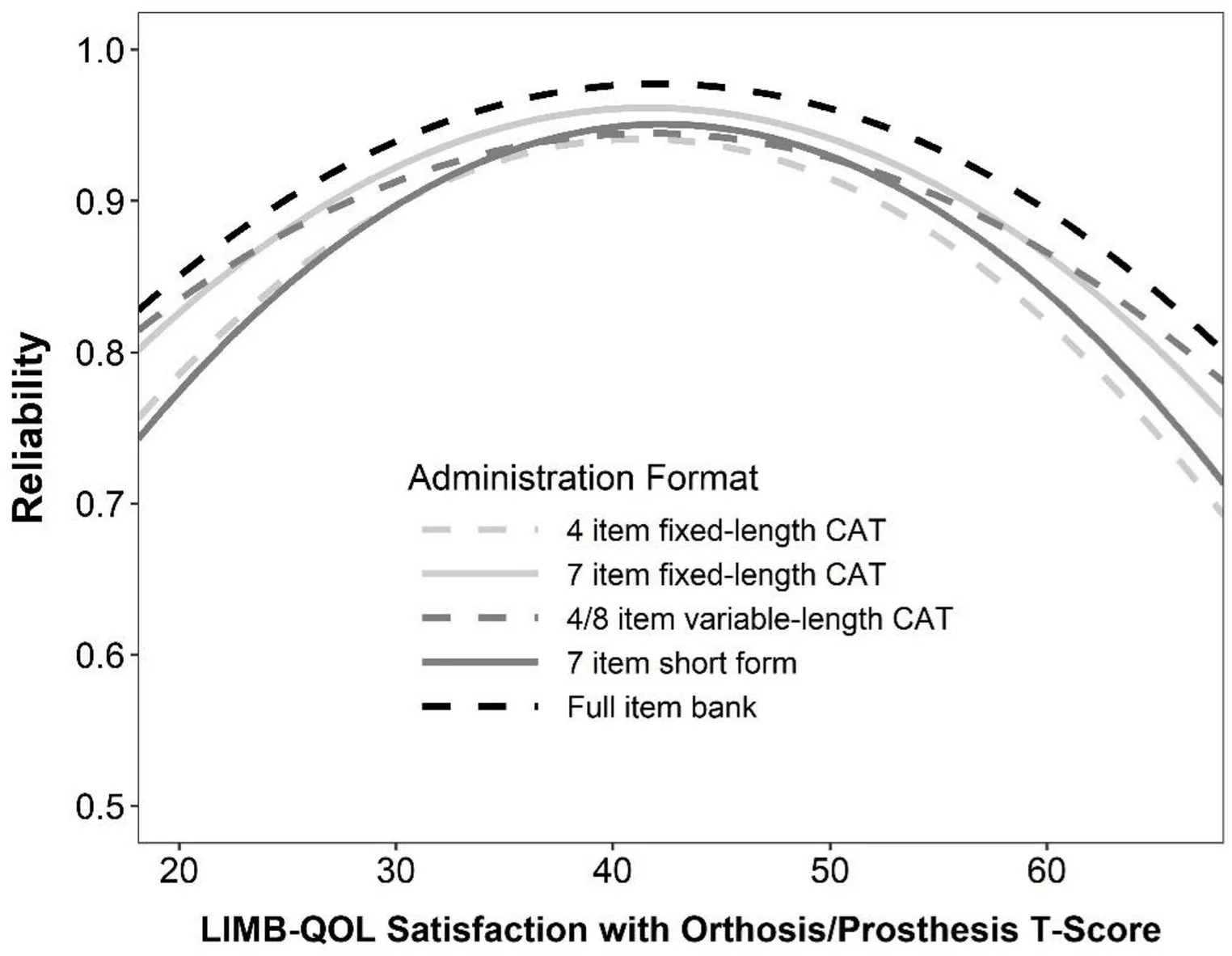
Classical reliability estimates across the latent trait continuum for LIMB-QOL Satisfaction with Orthosis/Prosthesis. Several different administration formats were simulated using real participant data, including the full 15-item bank, a 7-item fixed-length short form, and three CAT specifications (4-item fixed-length CAT, 7-item fixed-length CAT, 4-item minimum, 8-item maximum variable-length CAT). Reliability was highest for an approximate T-score equivalent of 42. Reliability was generally comparable across formats and exceeded .80 over a 3 + SD range on the latent trait

Short form and CAT versions were subsequently developed and compared to the full item bank. T-scores based on the item parameters were computed for the following abbreviated administrations: (a) a 7-item short form (SF7a), (b) a 4-item fixed-length CAT, (c) a 7-item fixed-length CAT, and (d) a variable-length CAT with at least 4 and at most 8 items administered, and a stoppage rule of < 0.3 standard errors. Descriptive summaries of the score distributions for these versions are shown in Table 5. Means and standard deviations were comparable across formats, as well as classical reliability, although the latter was highest for the full item bank and 7-item fixed-length CAT over most of the latent trait continuum (Fig. 2). Scores from the abbreviated versions were all strongly correlated with those from the full item bank.

**Table 5:** Score distributions and correlations with full item bank

Measure/Format	T-Score						Standard Error				Correlation with full bank (15 items)
	Mean	SD	Min	Max	Floor, %	Ceiling, %	Mean	SD	Min	Max	
4-item fixed-length CAT	49.9	9.1	25.0	64.7	2.1	10.7	3.5	1.0	2.3	5.8	0.96
7-item fixed-length CAT	49.9	9.4	21.6	66.8	0.9	7.1	2.9	1.0	1.9	5.5	0.99
Variable-length CAT (min 4 / max 8)	50.0	9.4	20.1	67.2	0.6	6.4	3.1	0.8	2.3	5.5	0.98
7-item short-form	50.0	9.3	22.9	65.3	0.9	10.3	3.3	1.1	2.2	5.8	0.98
Full item bank	50.0	9.7	18.1	68.2	0.6	5.6	2.5	1.0	1.6	5.3

### Reliability and validity

At the 1-year follow-up assessment, a short form of items was administered for test–retest reliability and validity analyses to reduce participation burden. This 4-item short form (SF4b) exhibited acceptable internal consistency reliability (α = 0.79, 95% CI = [0.76, 0.81]) and contained content representative of the full item bank. Over the 1- to 2-week retest period, test–retest reliability was good (ICC = 0.77, 95% CI = [0.69, 0.83]). Correlations between LIMB-QOL Satisfaction with Orthosis/Prosthesis and the criterion measures (TAPES-R, ABIS-R, PROMIS Global Health) are shown in Table 6. Four of the measures exceeded the pre-specified criterion of *r* > 0.40: TAPES-R General Adjustment (*r* = 0.59) and Social Adjustment (*r* = 0.46), ABIS-R (*r* = − 0.48), and PROMIS Global Health, Physical (*r* = 0.47). The other two measures included *r* > 0.40 in their 95% confidence intervals: TAPES-R Adjustment to Limitation (*r* =− 0.34) and PROMIS Global Health, Mental (*r* = 0.33). Finally, frequency of device use (see Table 3) was related to satisfaction scores at baseline for prosthesis users: individuals who reported using their device daily exhibited significantly higher satisfaction scores (M = 52.67) compared to those who used their device less than daily (M = 40.31; *t* [368] = 9.09, *p* < 0.01, *d* = 1.40, 95% CI = [− 1.71, − 1.08]). For orthosis users, the pattern was similar although the difference in scores between daily (M = 48.14) and less than daily users (M = 45.34) was less pronounced and not statistically different from zero (*t* [161] = 1.93, *p* = 0.054).

**Table 6:** Correlations between LIMB-QOL satisfaction with orthosis/prosthesis and criterion measures

			95% confidence interval	
Criterion measure	*n*	*r*	Lower limit	Upper limit
TAPES-R: general adjustment	145	0.59	0.47	0.68
TAPES-R: social adjustment	145	0.46	0.32	0.58
TAPES-R: adjustment to limitation	145	− 0.34	− 0.48	− 0.19
ABIS-R	144	− 0.48	− 0.59	− 0.34
PROMIS global health, mental	180	0.33	0.19	0.45
PROMIS global health, physical	180	0.47	0.35	0.58

TAPES-R,  Trinity amputation and prosthesis experience scales, Revised. ABIS-R  =  Amputee body image scale, Revised. PROMIS = Patient-reported outcomes measurement information system. All *p*s < .01

## Discussion

The Satisfaction with Orthosis/Prosthesis item bank, developed for the LIMB-QOL measurement system, is an innovative contribution to the field because it is not limited for use in a specific major extremity trauma sub-population. This is ideal for large-scale monitoring of this heterogeneous clinical population, where a mix of presentations involving multiple and single limb involvement, trauma and other sudden-onset illnesses, and upper- and/or lower-extremity damage are commonly treated all in one surgical or rehabilitation setting. The item bank development and calibration work closely followed the PROMIS standards for developing a CAT instrument and has yielded a bank of 15 high-quality items that span an array of relevant constructs under the domain of Satisfaction with Orthosis/Prosthesis. This measure adds to the available tools for evaluating and monitoring HRQOL outcomes associated with device satisfaction in people with limb loss and surgical repair/reconstruction, which could serve to improve the evidence base in the field of device prescription—a field that is affected by several challenges for precision rehabilitation and patient-centered care [10].

The correlations observed in this study between the new measure and existing measures support its convergent validity, while also suggesting that the new measure provides coverage of unique content. The pattern of these correlations indicates that general adjustment, amputee body image, physical health, and social adjustment are the topics most closely associated with the LIMB-QOL measure, although the magnitude of the correlations (all *r*s < 0.60) suggests incomplete overlap of content coverage. Device use frequency (daily vs. less-than-daily) was significantly related to satisfaction for prosthesis users, although this pattern did not reach statistical significance for orthosis users. This result further supports the construct validity of the new measure because it shows associations with real-world outcomes.

Research has shown that the effects of assistive devices on individuals with major extremity injuries are complex and include psychosocial components—including desire for independence, body image, community reintegration and participation in social roles—as well as the functional components measured by most outcome measures [106]. The LIMB-QOL is intended to account for the multifaceted influences on HRQOL after limb injury and loss. Satisfaction with Orthosis/Prosthesis is one component of this measurement battery that provides a standardized method for evaluating patient experience of satisfaction with their device(s). Based on the results of this study, we recommend it be used by researchers and clinicians seeking to document an individual’s experience of their everyday satisfaction with the use of one or more devices, including their performance and contribution to well-being. We believe this PROM could be useful for clinical research evaluating approaches or algorithms for device prescription, barriers to device adoption, as well as for device effectiveness trials or other studies designed to advance precision rehabilitation and patient-centered care [10].

### Study limitations and future directions

The item bank was calibrated, and the reliability and validity were evaluated, in a sample of young to middle aged adults with sudden-onset causes of limb differences. The item bank has not been tested in a sample of prosthesis and orthosis users with extremity injuries secondary to chronic illnesses such as diabetes leading to limb loss and prosthesis use, or other dysvascular or degenerative neuromuscular conditions leading to orthosis use. Further research is necessary to support validity for use in these populations. Similarly, the sample was predominantly male, and we recommend further evaluation in women to determine if there are gender-specific factors influencing satisfaction with assistive devices. Likewise, due to imbalances in the sample makeup, we were unable to formally test gender, race, and education with DIF analyses. Future research to evaluate DIF by these demographic factors is recommended. Finally, with regard to sample makeup, we note that the validation study subsample did not contain participants that solely used an orthotic device. However, because 18 individuals that used both types of devices were part of the subsample, we do not have reason to doubt the generalizability of the validation study results to orthotic device users, although this deserves confirmation in future research.

This research did not identify factors that were likely to predict high and low satisfaction, which would address the clinical validity of this measure. Another area for further study using this measure would be to evaluate if comorbid conditions such as traumatic brain injury and posttraumatic stress disorder, or co-occurring systems like pain and depression, potentially mediate or moderate device satisfaction, as has been suggested by previous research [52, 107–110].

## Conclusions

The LIMB-QOL Satisfaction with Orthosis/Prosthesis measure assesses an individual’s satisfaction with and emotional and physical assimilation of an orthosis and/or prosthesis. This includes satisfaction with device performance and aesthetics as well as user adaptation to a device, including usage, fit, and comfort. It is brief and reliable measure developed with advanced IRT methodology and shows evidence of validity for use to assess HRQOL after major extremity injury/sudden-onset limb-threatening illness.

## Electronic supplementary material

Below is the link to the electronic supplementary material.

Supplementary file 1 (DOCX 1 kb)
