# Supplementary material for: Measuring satisfaction with orthotic and prosthetic devices
Source: Qual Life Res. 2026 Aug 7;35(9):259. doi: 10.1007/s11136-026-04366-x (PMC13451311; doi:10.1007/s11136-026-04366-x)
Supplement: Supplementary file 1 [file 11136_2026_4366_MOESM1_ESM.docx]

# Supplemental Material

## Table S1. Validation Study Sample Demographics by Criterion Measure

|  | **TAPES-R** (*n* = 145) | | **ABIS-R**  (*n* = 144) | | **PROMIS**  **Global Health**  (*n* = 180) | |
| --- | --- | --- | --- | --- | --- | --- |
|  | ***M*** | **SD** | ***M*** | ***SD*** | ***M*** | **SD** |
| Age | 43.6 | 12.9 | 43.7 | 12.9 | 42.3 | 13.1 |
| Time Since Injury (Years) | 12.3 | 10.7 | 12.3 | 10.7 | 11.3 | 10.4 |
|  | **n** | **%** | **n** | **%** | **n** | **%** |
| Gender |  |  |  |  |  |  |
| Male | 112 | 77.2 | 111 | 77.1 | 140 | 22.2 |
| Female | 33 | 22.8 | 33 | 22.9 | 40 | 77.8 |
| Other | 0 | 0.0 | 0 | 0.0 | 0 | 0.0 |
| Do not wish to provide | 0 | 0.0 | 0 | 0.0 | 0 | 0.0 |
| Ethnicity |  |  |  |  |  |  |
| Non-Hispanic | 124 | 85.5 | 123 | 85.4 | 150 | 83.3 |
| Hispanic | 21 | 14.5 | 21 | 14.6 | 29 | 16.1 |
| Do not wish to provide | 0 | 0.0 | 0 | 0.0 | 1 | 0.6 |
| Race |  |  |  |  |  |  |
| American Indian / Alaskan Native | 1 | 0.7 | 1 | 0.7 | 1 | 0.6 |
| Asian | 2 | 1.4 | 2 | 1.4 | 3 | 1.7 |
| Black or African American | 9 | 6.2 | 10 | 6.9 | 14 | 7.8 |
| Native Hawaiian or Other Pacific Islander | 3 | 2.1 | 3 | 2.1 | 3 | 1.7 |
| White | 116 | 80.0 | 114 | 79.2 | 141 | 78.3 |
| Other | 7 | 4.8 | 7 | 4.9 | 9 | 5.0 |
| Do not wish to provide | 1 | 0.7 | 1 | 0.7 | 1 | 0.6 |
| Multiple | 6 | 4.1 | 6 | 4.2 | 8 | 4.4 |
| Injury Location |  |  |  |  |  |  |
| Upper | 20 | 13.8 | 19 | 13.2 | 30 | 16.7 |
| Lower | 102 | 70.3 | 103 | 71.5 | 124 | 68.9 |
| Both Upper and Lower | 23 | 15.9 | 22 | 15.3 | 26 | 14.4 |
| Limb Status ^a^ |  |  |  |  |  |  |
| Limb loss/Amputated | 140 | 96.6 | 142 | 98.6 | 144 | 80.0 |
| Surgically Treated | 5 | 3.4 | 2 | 1.4 | 36 | 20.0 |
| Device Use Status |  |  |  |  |  |  |
| Prosthetic Device User | 127 | 87.6 | 125 | 86.8 | 128 | 71.1 |
| Orthotic Device User | 0 | 0.0 | 3 | 2.1 | 35 | 19.4 |
| Both Prosthetic and Orthotic Device User | 18 | 12.4 | 16 | 11.1 | 17 | 9.4 |
| Trauma Status ^b^ |  |  |  |  |  |  |
| Traumatic | 117 | 80.7 | 115 | 79.9 | 149 | 82.8 |
| Non-Traumatic | 28 | 19.3 | 29 | 20.1 | 31 | 17.2 |
| Military Status |  |  |  |  |  |  |
| Active-Duty ^c^ | 18 | 12.4 | 19 | 13.2 | 34 | 18.9 |
| Veteran | 64 | 44.1 | 63 | 43.8 | 73 | 40.6 |
| Civilian | 63 | 43.4 | 63 | 43.8 | 73 | 40.6 |

*Notes:* Due to rounding, percentages within categories may not add to 100. ^a^ = Limb status of “Limb loss/Amputated” is defined as the number of people in the sample who had experienced the loss of at least one major extremity due to a qualifying cause, and “Surgically Treated” indicates the presence of no limb loss/amputations; ^b^ = Trauma status of “Traumatic” is defined as the number of people in the sample who had experienced at least one qualifying major extremity injury having occurred due to a traumatic cause, with “Non-Traumatic” indicating no limb injuries were due to a traumatic cause; ^c^ = Includes military reservists. TAPES-R = Trinity Amputation and Prosthesis Experience Scales-Revised. ABIS-R = Amputee Body Image Scale – Revised. PROMIS = Patient-Reported Outcomes Measurement Information System.

## Table S2. Raw Score to T-score Conversion Table for LIMB-QOL v1.0 Satisfaction with Orthosis/Prosthesis Short Form 7a

| **Raw Score** | **Scaled T-Score** | **Standard Error** |
| --- | --- | --- |
| 7 | 22.8 | 4.4 |
| 8 | 25.8 | 3.8 |
| 9 | 27.3 | 3.6 |
| 10 | 28.9 | 3.3 |
| 11 | 30.3 | 3.2 |
| 12 | 31.6 | 3.0 |
| 13 | 32.8 | 2.9 |
| 14 | 33.9 | 2.9 |
| 15 | 35.0 | 2.8 |
| 16 | 36.0 | 2.8 |
| 17 | 37.1 | 2.8 |
| 18 | 38.1 | 2.8 |
| 19 | 39.1 | 2.8 |
| 20 | 40.1 | 2.9 |
| 21 | 41.2 | 2.9 |
| 22 | 42.2 | 2.9 |
| 23 | 43.3 | 2.9 |
| 24 | 44.4 | 2.9 |
| 25 | 45.5 | 3.0 |
| 26 | 46.7 | 3.0 |
| 27 | 47.9 | 3.0 |
| 28 | 49.1 | 3.1 |
| 29 | 50.5 | 3.2 |
| 30 | 52.0 | 3.4 |
| 31 | 53.7 | 3.6 |
| 32 | 55.5 | 3.8 |
| 33 | 57.8 | 4.1 |
| 34 | 60.6 | 4.6 |
| 35 | 65.4 | 5.8 |
